# Intestinal Pseudo-Obstruction After Vincristine Use

**DOI:** 10.7759/cureus.74213

**Published:** 2024-11-22

**Authors:** Miguel Monteiro, Beatriz Castanheira, Elsa Meireles, Nuno Príncipe, José-Artur Paiva

**Affiliations:** 1 Department of Intensive Care Medicine, Unidade Local de Saúde do Tâmega e Sousa, Penafiel, PRT; 2 Department of Oncology, Unidade Local de Saúde de Almada/Seixal, Almada, PRT; 3 Department of Intensive Care Medicine, Unidade Local de Saúde de Entre Douro e Vouga, Santa Maria da Feira, PRT; 4 Department of Intensive Care Medicine, Centro Hospitalar Universitário de São João, Porto, PRT; 5 Department of Medicine, Faculty of Medicine, University of Porto, Porto, PRT

**Keywords:** chemoinduced neuropathy, lymphoma, ogilvie's syndrome, paralytic ileus, vincristine

## Abstract

A 73-year-old man presented with nausea, abdominal discomfort, and distention persisting for the past five days. He had previously been diagnosed with stage III peripheral CD4+ T cell lymphoma and had initiated chemotherapy comprising vincristine two weeks prior to presentation. An evaluation revealed diffuse colon distention and pneumatosis intestinalis without mechanical obstruction, consistent with acute colonic pseudo-obstruction. The patient underwent surgical intervention and was subsequently admitted to the Intensive Care Unit due to distributive shock. Vincristine-induced ileus followed by intestinal pseudo-obstruction was suspected to be the underlying cause after excluding alternative causative factors.

## Introduction

Vincristine is a vinca alkaloid commonly employed in the treatment of various solid tumors and hematological malignancies [1]. The antimitotic drug exerts its anti-neoplastic effect by inhibiting microtubule polymerization and its application is constrained by a range of side effects, notably related to neurotoxicity [2].

Patients undergoing vincristine treatment typically exhibit varying degrees of neuropathy affecting both the somatic and autonomic peripheral nervous systems, a phenomenon intricately linked to dosage levels, with manifestations that may include symptoms such as paresthesia, numbness, orthostatic hypotension, and gastrointestinal complications like constipation, paralytic ileus and Ogilvie’s syndrome, which can potentially lead to intestinal ischemia and perforation [3]. While neurotoxicity is commonly acknowledged as a frequent side effect of vincristine treatment, there is limited understanding of its true incidence due to inconsistencies in its reporting, which stem in part from variations in assessment methods, study populations, and the lack of a gold standard for evaluating vincristine-induced peripheral neuropathy [3-5].

We present a case of vincristine-associated intestinal pseudo-obstruction in a 73-year-old male patient with stage III peripheral CD4+ T cell lymphoma. 

## Case presentation

A 73-year-old male, diagnosed with stage III peripheral CD4+ T cell lymphoma, presented to the emergency care department with persistent abdominal discomfort and distention over the preceding 5 days. Additionally, he reported symptoms of nausea, vomiting, and reduced stool volume. Upon clinical examination, the patient exhibited a tympanic and distended abdomen, accompanied by generalized pain. Despite maintaining orientation, his complexion appeared pallid, and signs of dehydration were evident, albeit with stable vital signs.

Thirteen days prior to admission, the patient underwent his inaugural cycle of chemotherapy, consisting of vincristine, cyclophosphamide, doxorubicin, etoposide, and prednisone. A recent PET scan revealed no evidence of intestinal lymphoma infiltration.

His medical history encompassed hypertension, dyslipidemia, obesity, and benign prostatic hypertrophy. Ambulatory pharmacotherapy included indapamide, dutasteride/tamsulosin, rifampicin, valaciclovir administered daily, and sulfamethoxazole/trimethoprim administered thrice weekly.

Laboratory results revealed a hemoglobin level of 12.2 g/dL, a white cell count of 4400/mm3 with normal differential count, and a platelet count of 117 000/mm3. Biochemical parameters showed hyponatremia of 106 mmol/L and hypokalaemia of 3.0 mmol/L with decreased serum osmolality of 231 mOsm/Kg and normal urinary osmolality of 658 mOsm/Kg and urinary sodium of 29 mmol/L. His C-reactive protein was 73.3 mg/L.

**Figure 1:**
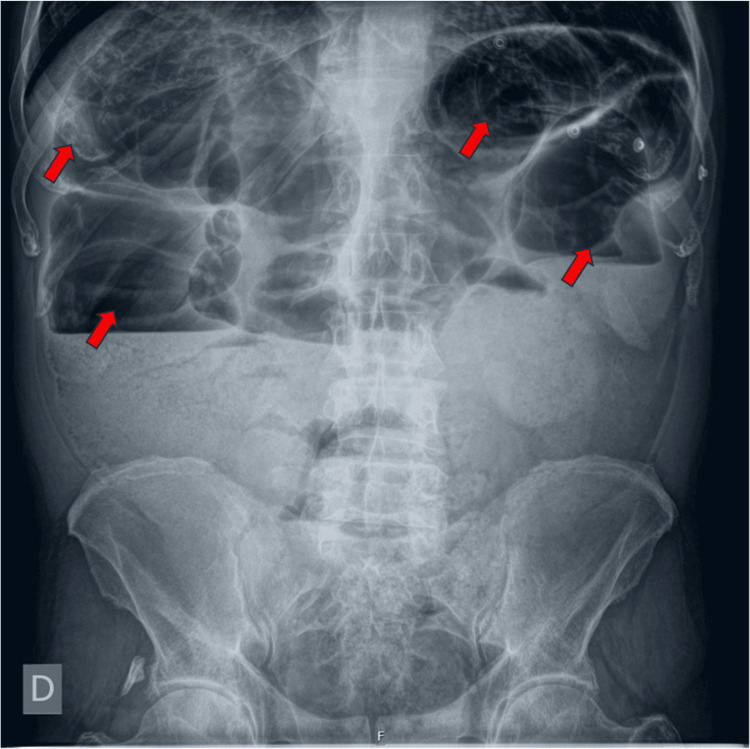
Abdominal radiograph depicting multiple air-fluid levels (red arrows) D – right side of the patient's body, F – foot end of the patient's body

The abdominal radiograph as shown in Figure 1 revealed multiple air-fluid levels.

**Figure 2:**
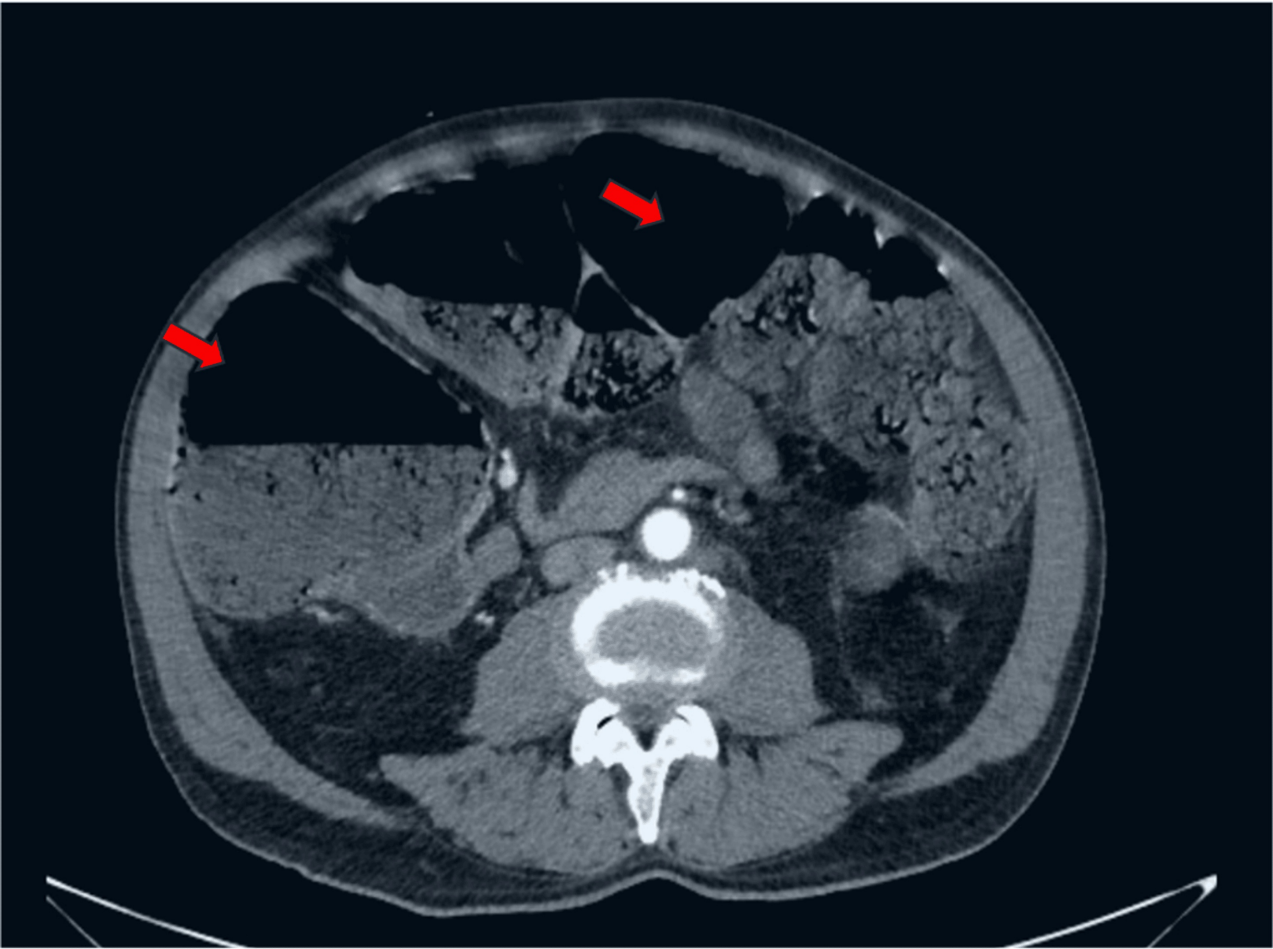
CT scan, axial plane of the abdomen showing bowel dilatation (red arrows)

Computed tomography (CT) of the abdomen depicted in Figure 2, revealed diffused dilatation of the ileum and colon with maximum diameters of 3.7 cm and 11.2 cm respectively, with suggestive signs of pneumatosis intestinalis in the portion of the ascending colon. CT also exhibited abnormal colon wall enhancement, suggesting ischemia. No signs of mechanical obstruction or vascular occlusion were noted. 

The patient underwent urgent laparotomy, during which a rectal decompression tube was inserted, yielding minimal clinical improvement. Laparotomy revealed dilatation of the small intestine and colon, with no obstructing anatomical lesions. Additionally, there were signs of irreversible ischemic damage to the cecum and colon. Subsequently, a right-sided hemicolectomy and appendicectomy were performed, along with the placement of an ileostomy and transverse colon mucous fistula.

After surgery, the patient was admitted to the intensive care unit due to distributive shock which we believe was caused by intestinal bacterial translocation associated with intestinal obstruction. Subsequently, multiorgan dysfunction ensued, necessitating analgo-sedation, mechanical ventilation, and vasopressor support. Empirical treatment with piperacillin/tazobactam and vancomycin was administered, resulting in clinical improvement. Peritoneal liquid and blood cultures were negative.

Peritoneal fluid cytology collected during surgery yielded negative results for malignant cells, while histopathology of the colon revealed an ischemic lesion devoid of malignancy. Consequently, the initiation of the second chemotherapy cycle was deferred until the resolution of the acute disease. During the hospital stay, the patient also reported peripheral paraesthesia. Vincristine was consequently discontinued from the chemotherapy regimen, which was resumed 23 days after admission.

## Discussion

Vincristine, a chemotherapy drug classified under vinca alkaloids, exerts its pharmacological action by binding to tubulin, thereby inhibiting microtubule polymerization, leading to mitotic disruption and subsequent cell apoptosis. While the exact pathogenesis of vincristine-induced peripheral neurotoxicity remains incompletely elucidated, several mechanisms have been proposed [1,6,7]. Vincristine's interaction with microtubules contributes to axonal and dendritic deformities, altered impulse transmission, and abnormal oligodendrocyte myelination, ultimately culminating in neuronal demise. Furthermore, vincristine appears to impact nerve cell mitochondria and endothelium, precipitating Wallerian degeneration and synaptic alteration [8].

The occurrence and severity of vincristine-induced peripheral neuropathy appear to be dose-dependent and cumulative, with manifestations encompassing sensory, motor, and autonomic neuropathies [6,7]. Globally and in the absence of pre-existing medical conditions the incidence of vincristine-induced neuropathy can reach as high as 70%, particularly among lymphoma patients [9]. 

Legha documented that the neuropathy usually appears within a couple of weeks of treatment initiation and their severity has been strongly linked with other factors, including patient characteristics, administration setup, pharmacokinetics, and genetic predispositions [10].

The triazole antifungal agents, commonly used for prophylaxis and treating invasive fungal infections during invasive chemotherapy, interact with the enzymes responsible for vincristine metabolism and thus potentiate the latter’s side effects [11].

Ogilvie's syndrome, first described by Sir William Ogilvie in 1948, refers to acute colonic pseudo-obstruction in the absence of any underlying mechanical obstruction. The exact pathophysiology remains unclear, but it is thought to result from disruptions in colonic reflex pathways, intrinsic dysfunction of the colon, chronic medical conditions, or pharmacological and metabolic disturbances [12].

Although colonic pseudo-obstruction secondary to autonomic neuropathy induced by vincristine is rare in adults, a handful of cases have been documented in the literature. For instance, Pessôa et al. reported a case of Ogilvie's syndrome in a 33-year-old female following vincristine-containing chemotherapy [13]. In this case report the authors describe the main findings of pseudo-obstruction being dilation extending from the cecum to the transverse colon and a transition zone in the splenic flexure, with a smaller caliber of the adjoining loop. The patient was submitted to surgery due to the risk of cecal perforation. Similarly, Geelen et al. documented three cases of Ogilvie's syndrome post-vincristine therapy, with one patient requiring urgent surgical intervention due to intestinal perforation, while neostigmine exhibited efficacy in inducing peristalsis in the remaining cases after conservative treatment failure [7]. Additionally, Diezi et al. proposed a management algorithm for vincristine-related intestinal pseudo-obstruction in children based on their review of 21 reported pediatric cases in the literature and description of five cases of pseudo-obstruction due to vincristine use in children [14].

Guidelines endorsed by American and European authorities advocate conservative measures as the primary approach in managing acute pseudo-obstruction [15-17]. This entails discontinuation or dose reduction of medications known to influence gastrointestinal motility, including vincristine. Should conservative measures fail, endoscopic decompression or neostigmine administration may be considered. Surgical intervention is warranted in the presence of fever, leukocytosis, abdominal tenderness, or radiological evidence of ischemia or perforation, particularly if cecal diameters exceed 12 cm as it is associated with an increased risk for complications [15].

Our patient presented clinical features consistent with vincristine-induced pseudo-obstruction, including progressive abdominal distension, discomfort, diminished stool volume, nausea, and vomiting, which manifested eight days following the initial vincristine dose. Additionally, the patient developed peripheral paraesthesia, further strengthening the causal relationship between vincristine treatment, neuropathy, and ileus. Electrolyte imbalance may have acted synergistically with vincristine in the etiology of ileus, possibly impairing gut motility via effects on ion channels, membrane potentials, and intracellular functions, however, the direct causal relationship between electrolyte derangements and the extent of ileus remains elusive [18,19]. Nevertheless, management guidelines for ileus recommend the correction of sodium, potassium, magnesium, and calcium [19]. Moreover, hyponatremia as seen in this case, is linked with intestinal wall ischemia and perforation serving as a predictor of severity [20]. We also found that the hyponatremia observed was likely associated with the syndrome of inappropriate antidiuretic hormone secretion which has also been linked to vincristine treatment [3].

Importantly, the patient had not received any other medications known to affect gastrointestinal motility, nor did he have underlying medical conditions that could impact colonic motor activity, such as disorder of the peripheral nervous system (e.g., neuropathy associated with diabetes, malnutrition, alcoholism, old age or inherited neuropathy), endocrinological diseases or *Clostridium difficile* infection. Additionally, mechanical causes such as tuberculosis or malignant infiltration of the intestine were excluded.

The suspicion of colonic pseudo-obstruction arose based on imaging findings demonstrating diffuse dilatation of the colon. Subsequently, signs indicative of ischemia and peritonitis prompted urgent surgical intervention, which ultimately confirmed the diagnosis.

## Conclusions

Vincristine-induced autonomic neuropathy presents with a spectrum of clinical manifestations, ranging from peripheral paresthesia to severe gastrointestinal complications such as Ogilvie's syndrome, as seen in this case. The neurotoxic effects of vincristine are dose-dependent, which limits the maximum tolerable dose and impacts its therapeutic use. A deeper understanding of the mechanisms underlying vincristine neurotoxicity could improve strategies for preventing and managing these adverse effects, potentially enhancing patient outcomes. In cases of colonic pseudo-obstruction, surgical intervention becomes necessary if there is evidence of ischemia, perforation, or if the cecal diameter exceeds 12 cm, to prevent life-threatening complications.
